# Simultaneous occurrence of accelerated nodulosis in lungs, liver, and kidneys, and acute exacerbation of interstitial pneumonia in a patient with rheumatoid arthritis: an autopsy case report

**DOI:** 10.1186/s12890-021-01806-x

**Published:** 2022-01-05

**Authors:** Akitake Suzuki, Shigeki Morita, Miho Ohshima, Nobuyoshi Minemura, Takeshi Suzuki, Masanobu Yoshida, Rikuo Machinami, Shuji Sakai, Chikao Torikata

**Affiliations:** 1 Department of Rheumatology, Mitsui Memorial Hospital, 1 Kanda-izumi-cho Chiyoda-ku, Tokyo, 101-8643 Japan; 2 Center for Rheumatology and Joint Surgery, Kawakita General Hospital, Tokyo, Japan; 3 Department of Pathology, Mitsui Memorial Hospital, Tokyo, Japan; 4 Department of Pathology, Kawakita General Hospital, Tokyo, Japan; 5 Department of Radiology, Tokyo Women’s Medical University, Tokyo, Japan

**Keywords:** Accelerated nodulosis, Rheumatoid nodules, Interstitial pneumonia, Acute exacerbation, Organizing diffuse alveolar damage, Rheumatoid arthritis, *Cryptococcus neoformans*

## Abstract

**Background:**

Accelerated nodulosis (ARN) is a rare variant of rheumatoid nodules (RNs) that is characterized by a rapid onset or the worsening of RNs. It generally develops at the fingers in patients with rheumatoid arthritis (RA) receiving methotrexate (MTX). Few case reports have described ARN at an extracutaneous location.

**Case presentation:**

An elderly patient with long-standing RA was admitted to our hospital with acute respiratory failure. Computed tomography upon admission showed diffuse ground-glass opacities superimposed with subpleural reticular shadowing and honeycombing and multiple nodules in the lungs and liver. Despite the discontinuation of MTX and introduction of an immunosuppressive regimen with pulse methylprednisolone followed by a tapering dose of prednisolone and intravenous cyclophosphamide, the patient died due to the acute exacerbation (AE) of RA-related interstitial lung disease (ILD) following the parallel waxing and waning of a diffuse interstitial shadow and pulmonary and liver nodules. At autopsy, RNs were scattered throughout both lung fields in addition to extensive interstitial changes. RNs were also detected in the liver and kidneys. The foci of cryptococcosis were mainly identified in alveolar spaces. Based on the clinical and pathological findings, these nodules were most consistent with ARN because of acute increases in the size and number of previously detected pulmonary nodules.

**Conclusion:**

The present case is noteworthy because ARN was concurrently detected in multiple internal organs and may be associated with the AE of RA-related ILD.

## Background

Classic rheumatoid nodules (RNs) occur in approximately 20 to 25% of patients with seropositive rheumatoid arthritis (RA). RNs develop as a later manifestation of active arthritic disease and are mostly identified in areas prone to mild repetitive traumatism, such as bony prominences, extensor surfaces, or regions adjacent to joints. Therefore, RNs commonly occur on the elbows and fingers, but rarely in the lungs or other internal sites. Risk factors for RNs are being male, smoking, ex-smokers, and elevated levels of serum rheumatoid factor (RF) and the anti-cyclic citrullinated peptide (anti-CCP) antibody [1, 2].

Accelerated nodulosis (ARN) is characterized by the rapid onset or worsening of RNs and appears to be a variant of RNs. It was originally recognized as a complication of methotrexate (MTX) therapy in patients with RA because lesions often regressed after the cessation of MTX and recurred after a re-challenge [3]. However, other drugs have been shown to induce ARN [1, 2]. Therefore, ARN is not merely a complication of MTX, it may also be associated with diverse risk factors, including previously unidentified factors.

We herein introduce a case of the simultaneous occurrence of ARN in the lungs, liver, and kidneys in addition to the AE of the usual interstitial pneumonia (UIP) subtype of RA-related ILD in a 74-year-old male with long-standing, anti-CCP antibody-positive RA.

## Case presentation

**Fig. 1:**
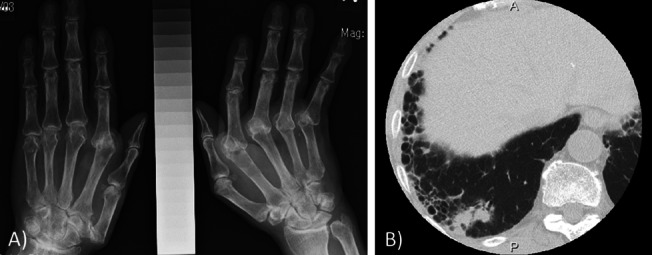
Radiological findings obtained at another hospital before admission. **A** X-ray of the hands obtained 13 months before admission showed joint space narrowing at the radiocarpal joint and intercarpal joints of the left hand. Subluxation at multiple sites of the metacarpophalangeal joints was also noted in both hands. **B** Chest CT taken 11 months before admission revealed a solitary nodule in the right lower lobe. Mild fibrosis showing subpleural honeycombing was observed in the same area

A 74-year-old male was transferred to our hospital with acute respiratory failure. He had been diagnosed with RA at the age of 57 years and had been treated with 12 mg per week of MTX, 1000 mg of sulfasalazine, and 100 mg of bucillamine at another hospital. RA disease activity had been maintained at a low level according to composite indices: the most recent simplified disease activity index and clinical disease activity index were 9.28 and 5.4, respectively. Previous radiography of the hands approximately 13 months before admission showed ulnar deviation of the left fingers as well as joint-space narrowing of the radiocarpal joint of the right hand (Fig. 1A). Chest computed tomography (CT) 11 months previously showed two solitary nodules in the right lower lobe and mild subpleural honeycombing in both lung fields (Fig. 1B). His previous medical history was significant for bladder cancer at the age of 56 years, which was treated by surgical resection and had not recurred thereafter. He also underwent arthroplasty of the left wrist at the age of 70 years and developed RA-related organizing pneumonia (details unknown). He was an ex-smoker (60 pack-years) with no environmental exposure. He suddenly developed breathing difficulties following a sore throat and fever of 39.0 °C and was transferred to our hospital.

On admission, body temperature was 39.6 °C, blood pressure 117/78 mmHg, and heart rate 130 beats per minute. Oxygen saturation while breathing 6 L of oxygen through a reservoir mask was approximately 92%. Pulmonary crackles were audible in whole lung fields. Joint deformities were noted at the bilateral fingers and wrists, while neither swelling nor tenderness was evident in any joint.

A laboratory examination upon admission was shown in Table 1. Our patient’s RF and anti-CCP antibody levels were 60.0 IU/ml (< 15 IU/ml) and 75.7 U/ml (< 4.4 U/ml), respectively. Serum Krebs von den Lungen-6 and surfactant protein-D levels were 930 U/ml (< 499 U/ml) and 33.0 ng/ml (< 109.9 ng/ml), respectively. His ß-D glucan value was 12.9 pg/ml (< 20.0 pg/ml) and the aspergillus and cryptococcus antigens were both negative. An arterial blood gas analysis (6 L of oxygen through a reservoir mask) showed a pH of 7.539, PCO_2_ 30.9 mmHg, PO_2_ 80.4 mmHg, HCO_3_ 25.8 mmol/L, and oxygen saturation 95.6%. Smears and cultures from sputum specimens for bacteria and mycobacterium were negative. Blood cultures did not yield any pathogen including bacteria and fungi. A polymerase chain reaction from the same specimens for tuberculosis, *Mycobacterium avium* complex, and *Pneumocystis jirovecii* were also all negative.

**Table 1:** Blood test results upon admission

Laboratory findings		Criterion value
WBC (/µL)	9100	3500 ~ 8500
Neu (%)	84.2	28.0 ~ 77.0
Lymp (%)	8.2	17.0 ~ 57.0
Mono (%)	7.3	0.0 ~ 10.0
Eosino (%)	0.0	0.0 ~ 10.0
RBC (/µL)	308 × 10*4	410 × 10*4 ~ 530 × 10*4
Hb (g/dL)	10.6	14.0 ~ 18.0
Hct (%)	30.6	36.0 ~ 48.0
Plt (/µL)	17.2 × 10*4	15.0 × 10*4 ~ 35.0 × 10*4
ESR (mm/hr)	120 <	4 ~ 18
TP (g/dL)	5.87	6.7 ~ 8.3
Alb (g/dL)	2.28	3.90 ~ 4.90
AST (IU/L)	44	7 ~ 38
LDH (IU/L)	8	4 ~ 36
ALP (IU/L)	225	120 ~ 370
Cr (mg/dL)	0.98	0.60 ~ 1.00
BUN (mg/dL)	26.6	8.0 ~ 20.0
CRP (mg/dL)	21.36	0 ~ 0.3
RF (IU/ml)	60.0	0 ~ 15
Anti-CCP antibody (U/ml)	75.7	0 ~ 4.4
KL-6 (U/ml)	930	0 ~ 499
SP-D (ng/ml)	33.0	0 ~ 109.9
BNP (pg/ml)	14.3	0 ~ 18.4
β-d glucan (pg/ml)	12.9	0 ~ 20.0
Aspergillus antigen	Negative	Negative
Cryptococcus antigen	Negative	Negative

**Fig. 2:**
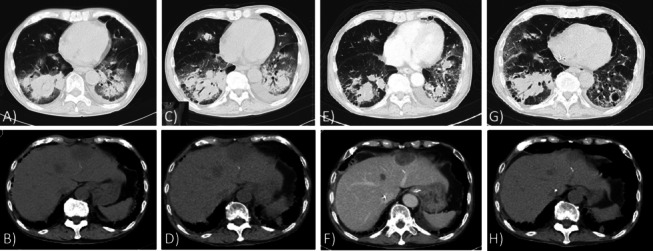
Radiological findings over time. **A**, **B** CT on admission revealed bilateral ground opacities superimposed with a subpleural reticular shadow and honeycombing. Multiple nodular lesions were detected in the bilateral lung fields and liver. **C**, **D** Re-examination of CT before intubation on the 4th hospital day showing the deterioration of opacities along with traction bronchiectasis. Pulmonary and liver nodules slightly increased in size. **E**, **F** On the 12th hospital day after weaning from the mechanical ventilator, diffuse ground-glass opacities improved and pulmonary and liver nodules decreased in size. **G**, **H** On the 21st hospital day before re-intubation, the interstitial shadow was exacerbated and the sizes of pulmonary nodules increased. Liver nodules showed no significant changes in size. Mediastinal emphysema was detected

Chest radiography showed diffuse ground-glass opacities and chest CT on admission revealed bilateral ground-glass opacities superimposed with a subpleural reticular shadow and honeycombing. Multiple nodular lesions were detected in the bilateral lung fields and liver (Fig. 2A), and ranged in size from 15 to 45 mm. All medications, including MTX, were suspended and combined antibiotic therapy, including ceftriaxone, piperacillin-tazobactam, and trimethoprim-sulfamethoxazole, was immediately initiated. However, respiratory failure was suddenly exacerbated on the 4th hospital day, and the patient was intubated and managed by mechanical ventilation. A re-examination of chest CT before intubation showed the deterioration of ground-glass opacities and the foci of traction bronchiectasis inside of lesions. Pulmonary nodules became larger than those detected on admission (Fig. 2B). Based on a diagnosis of the AE of RA-related ILD, pulse methylprednisolone was initiated at 1000 mg/day for 3 days, followed by a tapering dose of 60 mg/day of prednisolone and 750 mg of intravenous cyclophosphamide. Ultrasonography of the liver showed tumors in S3 (45 × 33 mm) and S4 (30 × 26 mm) and liver biopsy obtained on the 7th hospital day revealed extensive coagulation necrosis with granulation tissue, but did not lead to a definite diagnosis. On the 11th hospital day, the patient was successfully weaned from ventilatory support and extubated. Repeated chest CT, obtained at the 12th and 19th hospital days, confirmed synchronous improvements in the diffuse interstitial shadow and multiple pulmonary and liver nodules (Fig. 2C).

On the 21st hospital day, while taking 40 mg per day of prednisolone, respiratory failure suddenly worsened and the patient was reintubated and managed by mechanical ventilation. Chest CT revealed the deterioration of ground-glass attenuation and pulmonary nodules in the right lung field (Fig. 2D). Pulmonary lesions progressed despite a second course of methylprednisolone pulse therapy and intravenous cyclophosphamide with the addition of cyclosporine, and the patient died due to respiratory failure on the 39th hospital day. Blood cultures on the 39th hospital day later yielded *Cryptococcus neoformans*.

**Fig. 3:**
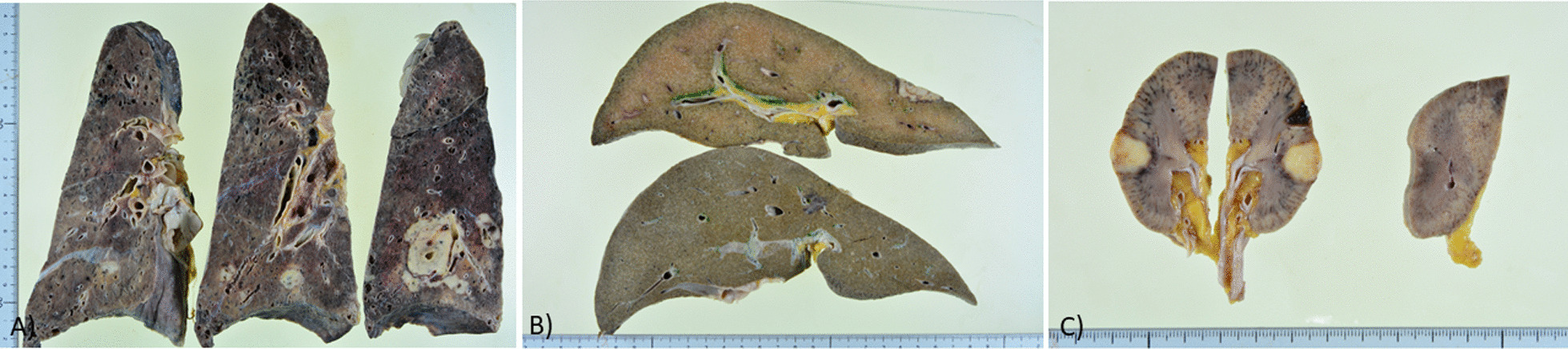
Macroscopic findings. **A** Cut surfaces of the bilateral lungs, which had a hard consistency and multiple white nodular consolidations, predominantly in the lower lobes. **B** Cut surface of the liver, showing white nodular consolidations, which were also diagnosed as rheumatoid nodules. **C** Cut surface of the kidney, showing white nodular consolidations, which were also diagnosed as rheumatoid nodules

**Fig. 4:**
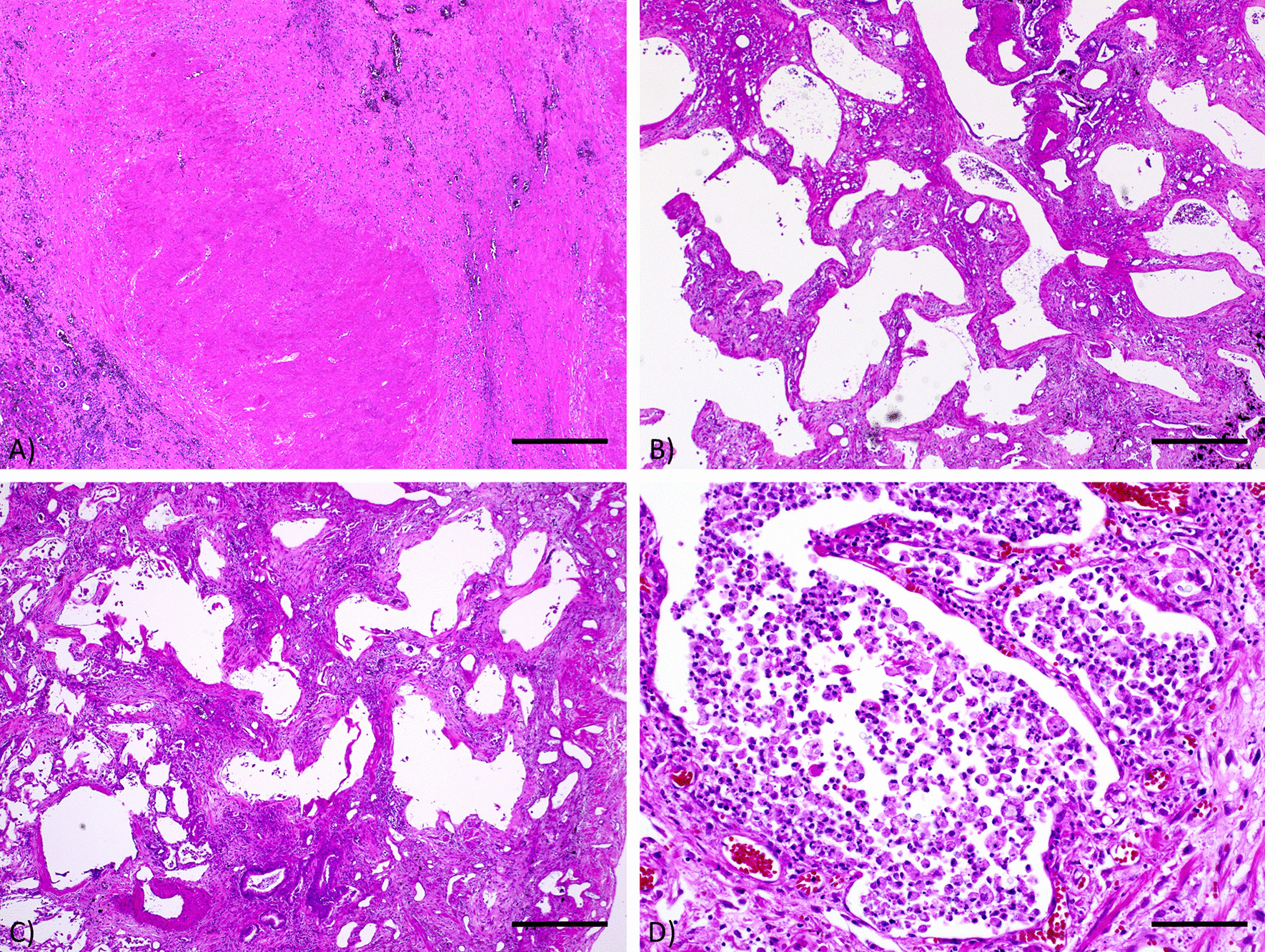
Microscopic findings. Hematoxylin and eosin　stained specimen (original magnification; **A**–**C** × 40; **D** × 200) were observed under OLYMPUS BX-53 microscope. Photos were captured through a CCD digital camera (Leica DFC295) and recorded by LAS software V4.12. The scale bar is 500 μm (**A**–**C**) or 100 μm (**D**). **A** A rheumatoid nodule of the liver. The nodule consisted of a focus of central necrosis surrounded by palisading granuloma. **B** The majority of alveolar septa adjacent to the pleura were thickened and densely fibrotic, which caused frequent subpleural honeycombing. **C** Interstitial changes composed of relatively mature fibrosis and fibroblastic proliferation along with moderate degree of mononuclear cells’ infiltrates. Note detaching alveolar epithelial cells from alveolar septa and focal squamous metaplasia. Those findings were consistent with organizing diffuse alveolar damage. **D** Cryptococcus pneumonia. The alveolar space was filled with round and pale organisms

At autopsy, the cut surfaces of both lungs were fibrotic and there were multiple nodules up to 40 mm in diameter predominantly at the peribronchial parenchyma in both lower lobes (Fig. 3A). There were also solitary nodules in liver and right kidney (Fig. 3B and C). Microscopically, multiple nodules in the lungs, liver, and kidneys consisted of a focus of central necrosis surrounded by a palisading granuloma (Fig. 4A), which was consistent with RNs. The majority of alveolar septa adjacent to the pleura were thickened and densely fibrotic, which caused frequent subpleural honeycombing (Fig. 4B). There were scattering areas of interstitial changes composed of relatively mature fibrosis and fibroblastic proliferation along with a moderate degree of mononuclear cell infiltration. Alveolar epithelial cells were detached from alveolar septa and squamous metaplasia was detected in a few areas, all of which were consistent with organizing diffuse alveolar damage (DAD) (Fig. 4C). A pathological diagnosis of organizing DAD on the back of UIP-like RA-related ILD was established. At a higher magnification, round and pale organisms, which were positive for Grocott’s staining, were mainly detected in alveolar spaces (Fig. 4D) in a relatively diffuse manner. They were found in the pulmonary parenchyma adjacent to one RN. Pathological findings were compatible with pulmonary cryptococcosis. Cryptococcal infection was not detected inside RNs or in other organs.

## Discussion and conclusion

The present case showed the simultaneous occurrence of multiple pulmonary and liver nodules during the AE of RA-related ILD. Since pulmonary nodules may cause significant differential diagnostic issues [4], we considered multiple pulmonary and liver nodules at presentation to be lung cancer, metastatic tumors from bladder cancer, an MTX-related lymphoproliferative disorder, and fungal or tuberculous infections. However, an autopsy examination revealed that these nodules showed necrotizing granulomas surrounded by a palisading granuloma. Neither *M. tuberculosis* nor a detectable fungus was detected in these granulomas in the lungs, liver, or kidneys. Furthermore, malignant cells were not present. Therefore, a pathological diagnosis of RNs was established.

RNs are considered to be the most characteristic extra-articular feature of RA. The sites that most commonly develop RNs are the elbows and fingers. However, atypical internal sites for RNs were previously shown to include the lungs, pleura, heart, pharynx, pericardium, liver, pancreas, and kidneys [1, 2, 5–8]. Risk factors for RNs are being male, smoking, ex-smokers, and elevated levels of RF and the anti-CCP antibody [1, 2]. Our patient was a male ex-smoker who tested positive for RF and the anti-CCP antibody. Chest CT 11 months before admission revealed two solitary nodules in the periphery of the right lower lobe along with interstitial changes predominantly in the peripheral areas. Autopsy showed no findings other than RNs that may explain the solitary nodules. Based on these findings, solitary nodules were RNs.

Upon admission, CT revealed multiple large nodules in the lungs. Solitary nodules had increased in size and there were also newly developed multiple nodules in the right middle lobe and both lower lobes as well as in the liver. These nodules were markedly larger in size despite the continuous suspension of MTX and showed waxing and waning over time.

ARN is characterized by the rapid onset or worsening of RNs and appears to be a variant of RNs. Each nodule of ARN is clinically and histologically indistinguishable from standard RNs [2]. A nodule of ARN was originally considered to be a complication of MTX therapy in patients with RA because lesions often regressed with the cessation of MTX and recurred after a re-challenge [3]. However, recent case reports have implicated biologic agents, such as etanercept and infliximab, in the development of ARN, and other drugs, including aromatase inhibitors and azathioprine, have also been associated with ARN [2, 3]. Due to the lack of clinical trials and paucity of complete case reports of ARN, it is difficult to elucidate the pathogenesis of ARN in each case; however, RNs will become ARN under diverse conditions, some of which have not yet been identified.

Based on the rapid increase observed in the size of previously detected pulmonary nodules, the formation of multiple nodules upon admission, which changed in size over the course of hospitalization, and the histological findings of necrotizing granulomas in the lungs, liver, and kidneys without the direct invasion of cryptococci at autopsy, these nodules were most consistent with ARN. A systematic review of ARN from 2002 comprising 58 cases from 3 case series revealed that 76% of cases had finger involvement [2]. No case report has described the simultaneous occurrence of ARN in non-cutaneous locations. Therefore, the present case is noteworthy because ARN was detected at three different internal sites during the same period.

ARN appeared to change in size in accordance with the severity of the diffuse interstitial shadow in both lung fields. Bilateral ground-glass opacities on chest CT were predominant around ARN. Therefore, we speculated that the progression of ARN was associated with the AE of RA-ILD. Since available case reports and clinical trials on ARN have been fragmented and incomplete, there is currently no information on whether the frequency of ARN is higher in RA with than without ILD and, thus, difficulties are associated with demonstrating our hypothesis. Nevertheless, we focused on two cases reports of concomitant fatal diffuse interstitial pneumonia and multiple RNs in patients with RA, a 57-year-old male from Japan and a 74-year-old male from Austria who died of respiratory failure, respectively, due to refractory diffuse pulmonary fibrosis despite intensive immunosuppressive interventions. Histological examinations of both patients revealed diffuse pulmonary fibrosis and numerous pulmonary RNs throughout the entire lung fields. In both case reports, some of the granulomas contained fibroblasts in their outer layers and the authors of both case reports inferred that the proliferation of fibroblasts in granulomas may have led to severe interstitial fibrosis [9, 10]. In the present case, an autopsy examination also showed organizing DAD in which the foci of fibroblast proliferation were detected, occasionally adjacent to some RNs. Therefore, we hypothesized that a causal link may exist between ARN and diffuse interstitial fibrosis. If this is the case, the triggers that transformed RNs into ARN remain inconclusive in the present case. Further studies are needed to clarify the triggers for ARN development as well as the relationship between ARN and RA-related ILD.

A blood culture obtained on the day of death yielded *C. neoformans* and autopsy showed cryptococcal infection in the right lower lobe. One of the main triggers for the AE of idiopathic interstitial pneumonias (IIPs) is infection [11]. The infection-induced AE of IIP accompanied respiratory symptoms, such as fever, a sore throat, and worsening dyspnea, all of which were observed in the present case. In one case report from Japan, the AE of IIP was triggered by *Aspergillus* empyema [12]. Therefore, cryptococcal pneumonia may have been the trigger for the AE of RA-ILD in the present case. However, cryptococcal infection was only detected later in the clinical course after the initiation of intensive immunosuppressive therapy, with initial negative results from serum antigen and blood cultures of the fungus on admission. We did not identify any relationship between cryptococcal infection and the AE of RA-ILD.

There were concerns that necrotizing granulomas were due to cryptococcosis rather than ARN. A previous case report described pulmonary cryptococcosis mimicking RNs in a patient with RA [13]. Similar to the present case, serum cryptococcal antigen and blood cultures of the fungus were negative upon admission when multiple pulmonary nodules were detected by chest CT. They showed waxing and waning within a short period of time without antimycotic agents. Autopsy findings revealed cryptococcal infiltrates in the lungs, in a relatively diffuse manner, predominantly in alveolar spaces. The foci of cryptococci were also detected adjacent to one granuloma, but not inside of it. There were no cryptococcal infiltrates in the liver or kidneys or in other organs. Given those findings, it was less likely that these nodules were due to cryptococcosis.

In summary, we herein present a male patient with long-standing RA who developed ARN in multiple internal organs. We speculate that the progression of ARN may have been associated with the AE of RA-ILD. Since there have not yet been any similar case reports, the present case will broaden the differential diagnoses of multiple pulmonary nodules, particularly nodules that show waxing and waning during the clinical course.

## Data Availability

All data and figures in this article are included in the manuscript and are available to the readers.

## Abbreviations

ARN: Accelerated nodulosis
RN: Rheumatoid nodule
MTX: Methotrexate
RA: Rheumatoid arthritis
AE: Acute exacerbation
ILD: Interstitial lung disease
RF: Rheumatoid factor
Anti-CCP: Anti-cyclic citrullinated peptide
UIP: Usual interstitial pneumonia
CT: Computed tomography
IIP: Idiopathic interstitial pneumonia
DAD: Diffuse alveolar damage

## References

[1] Garcia-Patos V (2007). Rheumatoid nodule. Semin Cutan Med Surg.

[2] Tilstra JS, Lienesch DW (2015). Rheumatoid nodules. Dermatol Clin.

[3] Kerstens PJ, Boerbooms AM, Jeurissen ME, Fast JH, Assmann KJ, van de Putte LB (1992). Accelerated nodulosis during low dose methotrexate therapy for rheumatoid arthritis. An analysis of ten cases. J Rheumatol.

[4] Kovacs A, Baksay B, Cserenyecz A, Molnar K, Takacs M, Szekanecz Z (2015). Occurrence of pulmonary rheumatoid nodules following biological therapies. Clin Rheumatol.

[5] Sekulic M, Weinblatt ME, Rennke HG (2019). Rheumatoid nodule formation in the kidney: a diagnosis of exclusion and a rare manifestation of rheumatoid arthritis involving the kidney. Kidney Int Rep.

[6] Usta IM, Uthman IW, Kattar M, Nassar AH (2010). Rheumatoid granuloma of the cervix and vagina: a challenging diagnosis and treatment. Obstet Gynecol.

[7] Schned AR, Moran M, Selikowitz SM, Taylor TH (1990). Multiple rheumatoid nodules of the renal cortex. Arch Intern Med.

[8] Smits JG, Kooijman CD (1986). Rheumatoid nodules in liver. Histopathology.

[9] Fellbaum C, Domej W, Popper H (1989). Rheumatoid arthritis with extensive lung lesions. Thorax.

[10] Kitamura A, Matsuno T, Narita M, Shimokata K, Yamashita Y, Mori N (2004). Rheumatoid arthritis with diffuse pulmonary rheumatoid nodules. Pathol Int.

[11] Kato M, Yamada T, Kataoka S, Arai Y, Miura K, Ochi Y (2019). Prognostic differences among patients with idiopathic interstitial pneumonias with acute exacerbation of varying pathogenesis: a retrospective study. Respir Res.

[12] Suzuki A, Kimura T, Kataoka K, Matsuda T, Yokoyama T, Mori Y (2018). Acute exacerbation of idiopathic pulmonary fibrosis triggered by Aspergillus empyema. Respir Med Case Rep.

[13] Jang DW, Jeong I, Kim SJ, Kim SW, Park SY, Kwon YH (2014). Pulmonary cryptococcosis that mimicked rheumatoid nodule in rheumatoid arthritis lesion. Tuberc Respir Dis (Seoul).

